# An Overview of Angle Deviations of Fiber-Reinforced Polymer Composite Angular Laminates

**DOI:** 10.3390/ma16134844

**Published:** 2023-07-05

**Authors:** Shun-Fa Hwang

**Affiliations:** Department of Mechanical Engineering, National Yunlin University of Science and Technology, 123 University Road, Section 3, Yunlin 64002, Taiwan; hwangsf@yuntech.edu.tw; Tel.: +886-5-534-2601 (ext. 4143)

**Keywords:** spring-back, spring-in, thermoset, thermoplastic, composite, finite element analysis

## Abstract

After manufacturing, fiber-reinforced polymer composite laminates may have residual stresses, resulting in warpage in flat structures and angle changes in angular sections. These shape distortions may cause fitting mismatch problems under high-level assembly, and extra efforts to fix these problems may be needed. The present paper only makes an overview of the angle deviation of angular composite laminates made of either thermoset matrix with autoclave curing or thermoplastic matrix with thermoforming. Depending on the positive or negative angle deviation, spring-back or spring-in behavior is observed. There are many parameters, including intrinsic and extrinsic parameters, that could affect the angle deviation. In the first part of this review paper, experimental results concerning the effects of the part angle, part thickness, lay-up sequence, corner angle, flange size, tool material, tool surface, and cure cycle are summarized. Spring-in angles are generally obtained in this part. In the second part, several prediction methods, such as simple equations and finite element methods, are compared to indicate the considered parameters. Some have good agreement and some have larger errors. The crucial differences may be dependent on the micromechanical theories and the input properties of the composite and the constituents.

## 1. Introduction

Fiber-reinforced polymer matrix composites are popular candidates for weight-critical structural components applied in the aerospace, automobile, sporting, and other industries because of their high values of strength/weight and stiffness/weight. Polymers are generally selected to be a matrix since they have low density and good bonding with fibers. Polymers can be classified as thermoset and thermoplastic ones, resulting in different properties and manufacturing processes for composite materials. Thermoset composite materials are popular because they have good mechanical properties, low forming temperature, low viscosity, and suitability for complicated component shapes. Thermoplastic matrix composites have recently gained attraction due to their fast processability, high impact strength, unlimited shelf life, and recyclability.

For the manufacturing of thermoset composite materials, curing is the main process used to solidify the prepreg into the desired shape via the application of heat and pressure. Autoclave processing, pultrusion, resin transfer molding, and filament winding are popular manufacturing methods. Autoclave curing is one of the high-quality manufacturing methods because of its robustness [1]. To reduce the equipment cost, one alternative to this is vacuum curing under a hot press. For thermoplastic composite materials, there is no curing process. However, due to their high viscosity, semi-finished products such as thermoplastic composite sheets are a better choice to use for further forming processes, for example, thermoforming, diaphragm forming, and hydroforming. For both thermoset and thermoplastic composites, temperature and pressure are two important factors during the manufacturing process. Therefore, residual stresses may be induced after the manufacturing process and cause the shape distortion of the final product [2,3,4,5,6]. For flat products, this is called warpage. For angular products, the concern is the angle deviation resulting in spring-in or spring-back behaviors [7].

The shape distortion of the final part may create fitting mismatch problems under high-level assembly [8], in which dimensional requirements are necessary. If it is press-fit to match the mating parts, a massive increase in the part’s internal residual stress level is induced. An alternative method to solve this mismatch problem is trimming, which is not easy to apply to these high-stiffness composite materials and may also cause the growth of the production cost, delivery time, and material waste. Furthermore, the residual stresses of the distorted part may lead to delamination, fiber waviness, matrix cracking, and wrinkling [9,10,11,12,13]. Therefore, it is always desired to minimize the shape distortion of the final part.

**Figure 1:**
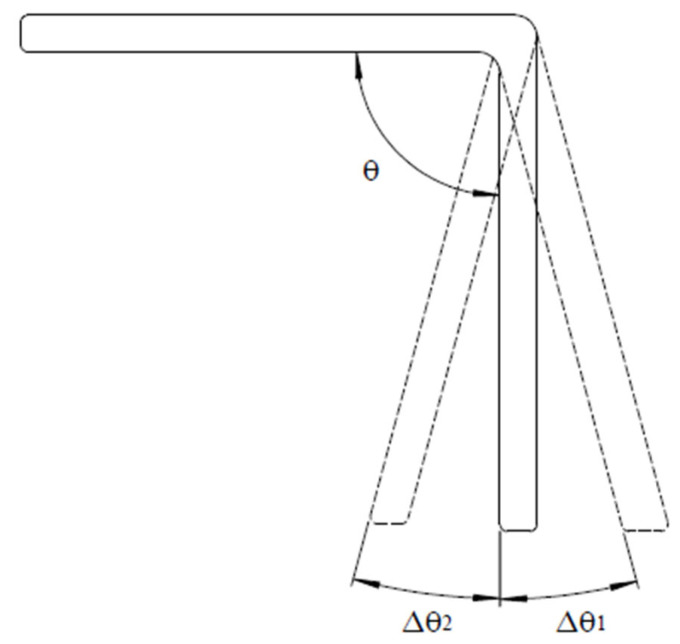
Angle deviation of the angular part.

**Figure 2:**
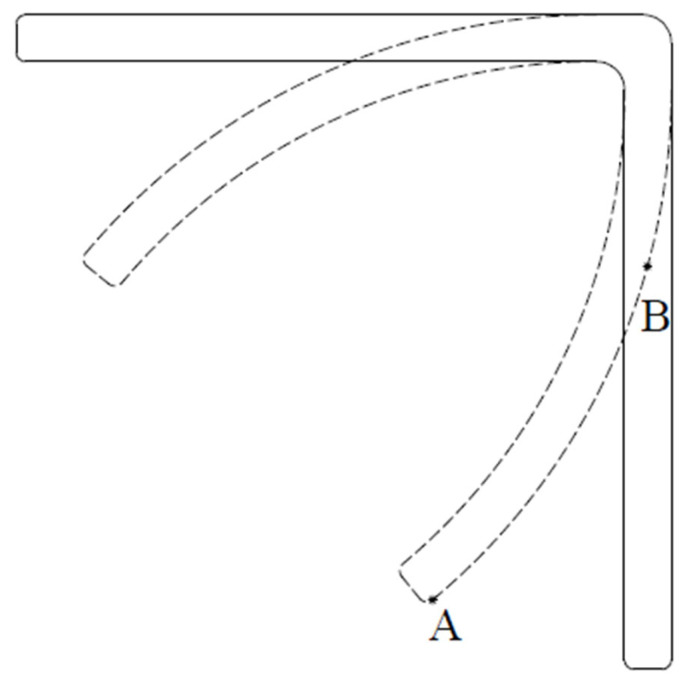
Selection difference of point A or B due to warpage of the flange.

When the final angle of the part is not the same as the tool angle, there are angle deviations in angular composite parts. When the enclosed angle of the part is larger than the tool angle θ or the desired angle, it is called the spring-back phenomenon and the angle deviation $\Delta \theta_{1}$ is positive, as shown in Figure 1. On the other hand, it is a spring-in behavior when the enclosed angle is less than the tool angle and the angle deviation $\Delta \theta_{2}$ is negative. Spring-back behavior generally implies the composite part wants to go back to its original shape after demolding, while spring-in behavior indicates the forward deformation of the composite part. In Figure 1, warpage of the flange is not considered, and it remains straight after manufacturing. If warpage of the flange [7,14] occurs, it will create measurement difficulties for the enclosed angle, as shown in Figure 2. There are different enclosed angles depending on which point is selected, point A or point B.

**Figure 3:**
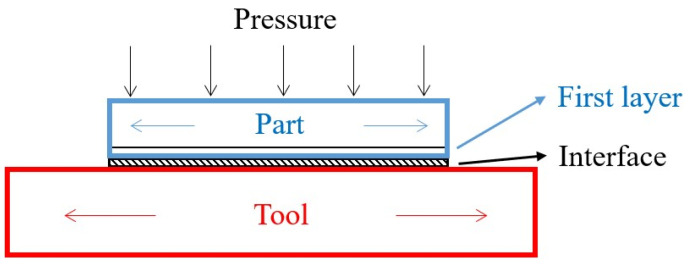
Interaction between the part and the tool.

Angle deviations of angular composite parts occur due to the development of residual stresses in the composite parts after the manufacturing process and demolding. There are so many parameters that could be considered to affect the residual stresses and the angle deviation. Generally, they are divided into intrinsic or extrinsic parameters [7]. Intrinsic parameters are those related to the composite parts, including the fibers, matrices, lay-up sequence, and part geometry. Extrinsic parameters are those concerning the manufacturing process and the tools. For example, the curing cycle, the tool materials, the tool surface conditions, and so on. In addition, there are interactions between the composite part and the tool [15,16], as shown in Figure 3. As shown, the expansion of the part and the tool differs during the manufacturing process, and this interaction will cause different expansions of the part along the thickness direction, yielding deformation of the part [17] or angle deviations [14].

**Figure 4:**
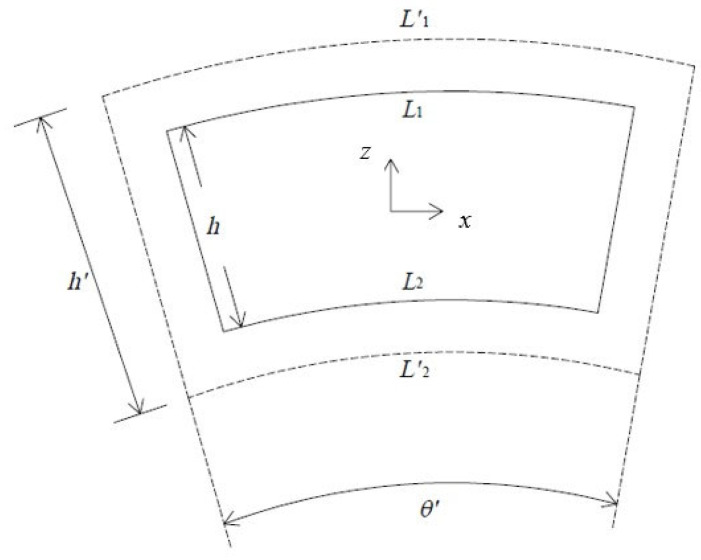
Elements before (solid lines) and after (dotted lines) expansion.

As shown in Figure 4, the orthotropic plate before deformation is represented by solid lines, and its dimensions are *L*_1_, *L*_2_, and *h*, the thickness. Before deformation, the original angle of the plate is $\theta =\left(L_{1}-L_{2}\right)/h$. After deformation, the plate is drawn in dotted lines. The two lines, *L*_1_ and *L*_2_, have elongated to $L_{1}^{\prime }$ and $L_{2}^{\prime }$. At the same time, the thickness has changed to $h^{\prime }$. Therefore, the final angle of the plate is $\theta^{\prime }=\left(L_{1}^{\prime }-L_{2}^{\prime }\right)/h^{\prime }$. If the deformation is due to thermal expansion, $L_{1}^{\prime }=L_{1}\alpha_{l}\Delta T$, $L_{2}^{\prime }=L_{2}\alpha_{l}\Delta T$, and $h^{\prime }=h\alpha_{t}\Delta T$. Here, the coefficients of thermal expansion (CTEs) along the longitudinal and transverse directions are denoted as $\alpha_{l}$ and $\alpha_{t}$, respectively. The temperature change is ΔT. Therefore, a simple equation to describe the angle deviation, Δθ, due to the anisotropy of the plate is as follows [7,18]:

(1) $$\Delta \theta =\theta^{\prime }-\theta =\theta \left[\frac{\left(\alpha_{l}-\alpha_{t}\right)\Delta T}{1+\alpha_{t}\Delta T}\right]$$

In addition to the effect of the CTE such as Equation (1), an equation proposed to describe the angle variation of angular composite parts by accounting for the hygroscopic and curing shrinkage effects is as follows [19,20]:

(2) $$\begin{array}{ll} \Delta \theta & =\Delta \theta_{CTE}+\Delta \theta_{HS}+\Delta \theta_{SK}\\ & =\theta \left[\frac{\left(\alpha_{l}-\alpha_{t}\right)\Delta T}{1+\alpha_{t}\Delta T}\right]+\theta \left[\frac{\left(\beta_{l}-\beta_{t}\right)\Delta C}{1+\beta_{t}}\right]+\theta \left[\frac{\emptyset_{l}-\emptyset_{t}}{1+\emptyset_{t}}\right] \end{array}$$

In this equation, $\Delta \theta_{CTE}$ denotes the angle deviation from the thermal effect. The angle deviation from the hygroscopic effect is $\Delta \theta_{HS}$, and the angle deviation from the shrinkage effect of the resin is $\Delta \theta_{Sk}$. The angle of the angular part is θ, and the moisture change is ΔC. Here, $\Delta \theta_{CTE}$ is the same as in Equation (1). By using the coefficient of hygroscope expansion along the longitudinal and transverse directions, $\beta_{l}$ and $\beta_{t}$, the term $\Delta \theta_{HS}$ can be similarly derived and obtained. The term $\Delta \theta_{Sk}$ can be obtained from the longitudinal shrinkage $\emptyset_{l}$ and the transverse shrinkage $\emptyset_{t}$, which are caused by the curing process or solidification of the resin.

The warpage of flat laminates after manufacturing has been intensively investigated in the literature [2,3,4,5,6,21,22], while the studies on the angle deviation of angular composite products are few. In addition, some results indicated spring-in behavior, while others showed the spring-back phenomenon. To attract attention, the present work tries to review the published articles about the angle deviation of angular products made of either thermoset composites with autoclave curing or thermoplastic composites with thermoforming. In the first part of this review paper, the measured angle deviations from the literature are collected and presented according to the matrix type and the affecting parameters, including intrinsic or extrinsic ones. In the second part, the simulation methods, which may be simple equations or finite element methods, from the literature used to predict the angle deviation of angular composite parts are mentioned and compared.

## 2. Parameter Effects from Experiments

Due to their popularity, thermoset composite materials are more studied than thermoplastic composite materials. For the process-induced deformation, the present paper focuses on thermoset composite angular parts made of carbon fiber/epoxy prepregs via an autoclave process, including the heat and the pressure. For thermoset polymer or epoxy, there is a curing process, in which the resin will experience viscous, rubbery, and glass stages. After the curing process, the resin is solidified, its volume is reduced, and residual stresses are created. Therefore, the chemical mechanism, thermo-mechanical mechanism, and the interaction between the composite part and the tool are the three main sources of the residual stresses in composite parts. From the viewpoint of intrinsic parameters, there are too many to be considered. Among them, the part angle, part thickness, flange size, corner radius, and lay-up sequence are commonly studied. The part angle means the desired enclosed part angle after the manufacturing process, as shown as θ in Figure 1, and it may also be called the tool angle. When the part angle is smaller, it means that the composite part undergoes a larger angle change during manufacturing. For this effect, Nasir et al. [14] concluded that the spring-in angle of the angular composite part from 1.4° to 0.2° was linear and inversely proportional to the part angle from 45° to 165°. When the part angle or the tool angle was smaller, larger residual stresses were created and the spring-in angle was larger [23]. Instead of the spring-in angle, Zakaria et al. [24] found that the spring-back value from 1.28° to 2.73° was inversely related to the part angle from 30° to 90° with different thicknesses and specimen sizes. Both papers showed that the angle deviation became larger as the part angle became smaller.

For the effect of the part thickness, Horberg et al. [25] revealed that the spring-in value decreased from 1.9° to 1.1° as the thickness of the L-shaped laminate was increased from 1 mm to 12 mm. A similar trend showing that thinner parts had larger spring-in values than thicker parts was obtained [7,20,26]. Ersoy et al. [27] indicated that as the thickness of C-sections made of unidirectional lay-ups was increased from 1 to 4 mm, the measured spring-in angles were decreased from 1.03° to 0.66°, which were expressed on the base of a 90° angle. In particular, Zakaria et al. [24] indicated that spring-back behavior was found for a 90° curved laminate and decreased with an increment of the part thickness. These studies showed that the angle deviation was reduced with the increase in the part thickness. The possible two reasons may be that thick laminates have higher bending stiffness and the interaction between the composite part and the tool may be limited to the two closest layers. However, some controversial results were found. For example, Stephan et al. [28] found that the spring-in value was increased by over 20% by doubling the part thickness. Patterson et al. [29] showed little effect of the part thickness. The possible reason for this controversial effect may come from multiple effects, including the composite part, the warpage, and the interaction between the composite part and the tool. In addition, these two references are old. Hence, the former conclusion about the effect of the part thickness should dominate the literature.

**Figure 5:**
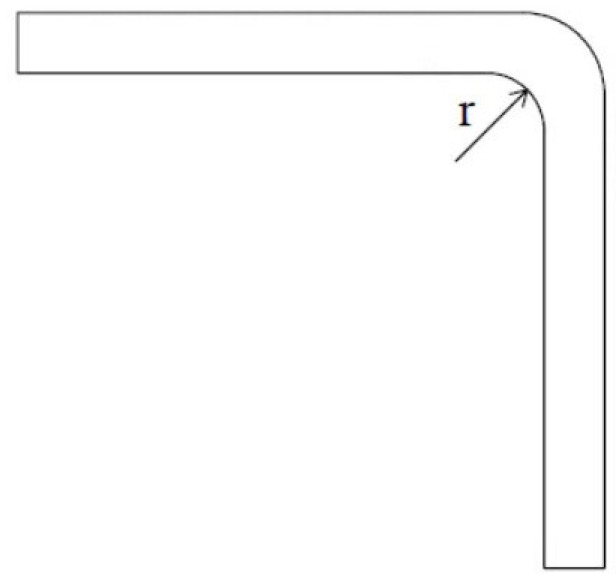
Corner radius of a part.

The lay-up sequence represents the change in the fiber angles among different layers. Considering U-shaped laminates with lay-ups of [0]_6_, [45]_6_, [90]_6_, and [0/90/0]_S_, Bellini et al. [30] found that the unidirectional 0° parts had about double spring-in angles as compared to the [90]_6_ part. The [0/90/0]_S_ part had the largest spring-in effect among the four lay-up parts. Ersoy et al. [27] indicated that the spring-in values for cross-ply C-shaped parts were larger than those for unidirectional 90° parts. The conclusion that quasi-isotropic parts had larger spring-in values than unidirectional 0° parts was confirmed by Albert et al. [7] for C-shaped composites and by Fernlund et al. [31] for L-shaped composites. Patterson et al. [29] showed little effect of the part lay-up for symmetrical laminates. It is believed that the lay-up sequence has a significant effect on the spring-in angle, and the explanation may be the through-thickness difference in the CTEs along the longitudinal directions. The corner radius means the fillet radius of the male tool or the angular part, as shown as *r* in Figure 5, and it is not related to the tool angle or part angle. For the effect of the corner radius, Bellini et al. [30] observed that when the corner radius of a U-shaped part was changed from 5 mm to 10 mm, the spring-in value was changed from 2.24° to 2.27°, indicating no obvious effect [29]. For the flange size of a 90-degree curved part, Zakaria et al. [24] changed it from 300 × 300 mm to 500 × 500 mm and the spring-back value was increased from 1.37° to 1.69°. Albert et al. [7] concluded that the spring-in angle increased when increasing the flange length. As for the part shape, comparing a C-shaped part with an L-shaped part, Fernlund et al. [31] indicated that the former had larger spring-in values than the latter, while Albert et al. [7] found little difference between these two shapes. To discuss the effects of the corner radius, flange size, and part shape, the warpage effect could not be neglected [7,30]. Considering the effect of the prepreg, Fernlund et al. [31] showed that a quasi-isotropic C-shaped part made of AS4/8552 prepregs had about 50% more spring-in values than that made of T800H/3900-2 prepregs. Horberg et al. [32] showed that wet L-shaped quasi-isotropic laminates with about 1.3–1.5% moisture weight had only approximately 35% of the spring-in angles of the same dry laminates, no matter the thickness.

**Figure 6:**
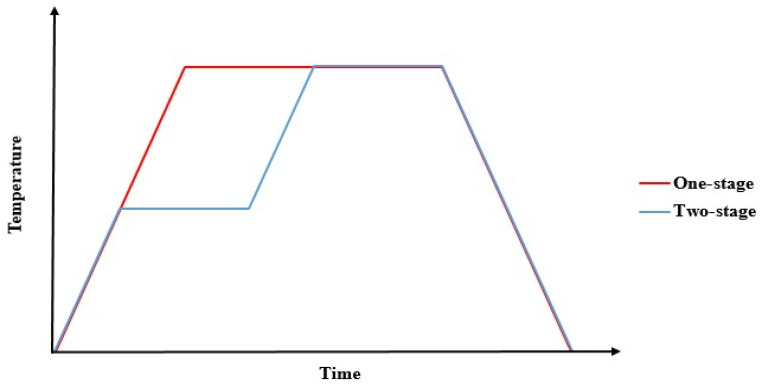
One-stage and two-stage curing cycles.

For the extrinsic parameters, the tool material, tool surface, and cure cycle were considered. Using different tool materials to make parabolic antenna reflectors, Shah et al. [33] concluded that an invar tool had less spring-back deformation as compared to other tool materials, including cast iron, stainless steel, and graphite, because of there being less difference in the coefficient of thermal expansion between invar and composite materials. Albert et al. [7] indicated that it was difficult to draw clear conclusions for tool materials between aluminum and steel. In some cases, aluminum tools had larger spring-in angles, while there was no difference in other cases. They argued that other parameters were interacting with the tool material. In addition, Kappel [34] argued that a mechanism called geometric locking at the corner of the angular parts may affect the interaction. The effect of the tool surface conditions, which may result in different slip conditions between the tool and the part, was considered by using different release agents. Fernlund et al. [31] indicated that a tool surface with a release agent and a fluorinated ethylene propylene (FEP) sheet had larger spring-in angles than that without an FEP sheet by a one-stage cure cycle. However, by a two-stage cure cycle, the former case had fewer spring-in angles than the latter. Albert et al. [7] obtained smaller spring-in angles for parts processed with a release agent and an FEP sheet. The effect of the cure cycle was generally investigated under a one-stage or two-stage temperature cycle, as shown by the red line or the blue line in Figure 6. The one-stage cure cycle made the gelation of the resin occur during this temperature stage. The two-stage cure cycle made the gelation occur before the second stage. The results that parts processed via a two-stage cycle had significantly larger spring-in angles than those processed via a one-stage cycle were verified by Albert et al. [7] and Fernlund et al. [31]. However, Shah et al. [33] concluded that the spring-back deformation under the one-stage cure cycle was about 5% lesser than that under the two-stage cure cycle for parabolic antenna reflectors. The effect of high curing pressure was shown to reduce the spring-in angles of L-shaped composite parts because of the thickness reduction [35]. Pereira et al. [26] observed that the spring-in angle of L-shaped laminates was decreased as a function of the time after autoclave fabrication because the cure needed to be complete. There are still other extrinsic parameters that were studied for the plate warpage, although not for the angle deviation. For example, the heating rate, cure temperature, cure time, and cooling rate are some parameters [36,37,38].

For angular thermoplastic parts, thermoforming processes are the main manufacturing methods. Generally, thermoplastic composite blanks were pre-heated to the desired temperature, which was generally higher than the melting temperature. Then, they were transferred into matched dies and formed into the desired shapes using pressure. Even though there is no curing process during these processes, there is still a volumetric change in the thermoplastic depending on the recrystallization situation, and residual stresses are induced. In addition to the intrinsic parameters, the extrinsic parameters, such as the forming temperatures and velocities, cooling rates, tool materials, and tool surface conditions, are important to the angle variation. However, only a few studies focused on the angle variation of angular thermoplastic parts. Salomi et al. [39] investigated the spring-in values of U-channel specimens made of E-glass fiber and polypropylene (PP) matrix by heating up to 180 °C and then fast cooling at 6.5 °C/min. The measured spring-in angle was 1.796° for a higher corner radius and 2.328° for a lower corner radius. This means a lower spring-in value for a higher corner radius. They also implied that the holding time or cooling rate of the thermoplastic composite at high temperatures had obvious effects on the spring-in behavior.

Padovec et al. [19] measured the angle deviation of L-sections made of carbon fiber/polyphenylene sulfide (PPS) thermoplastic composite with symmetrical lay-ups by heating the section to 100 °C and cooling it down to room temperature. Their results indicated there were 0.35–0.37% spring-backs, which were around 0.31–0.33 angle. Jain et al. [40] tried to design a tool to manufacture an aileron rib with the desired angles. The aileron rib was made of carbon fiber fabric-reinforced polyetherimide (PEI), which was an amorphous polymer and had a glass transition temperature of 217 °C. Their experimental results revealed that the final part angles were larger than the desired values, and this spring-back value could be decreased by increasing the tool temperatures. Engel et al. [41] formed a glass fiber-reinforced polyamide 6 into a 90-degree V-shape at 260 °C. Their results indicated that increasing the forming velocity from 0.1 to 1 mm/s, dwell time from 1 to 15 s, and forming pressure from 10 to 50 bars led to a decrease in the spring-in angles. Han et al. [42] indicated significant spring-in values from 2.087° to 3.431° for carbon fiber-reinforced PPS laminate thermoformed into a 92-degree V-shape as the forming temperature of 320 °C and the tool temperature changed from 80 °C to 230 °C. For symmetrical laminates, the lay-up sequence, either ${\left[{\left[\left(0/90\right)/\left(\pm 45\right)\right]}_{2}\right]}_{S}$ or ${\left[{\left[\left(\pm 45\right)/\left(0/90\right)\right]}_{2}\right]}_{S}$, had little effect on the spring-in value. Hwang et al. [43] investigated the angle deviation of woven carbon fiber/polycarbonate composites after thermoforming and found that the spring-in angle was around 2.60° for a 60° V-shape and around 1.72° for a 90° V-shape for ${\left[\left(\pm 45\right)\right]}_{6}$ blanks. The ${\left[\left(0/90\right)\right]}_{6}$ blank had a slightly higher spring-in angle than the previous blanks.

From the above experimental results, spring-in behavior is commonly presented in angular composite parts, and the discussed parameter effects from the literature listed in this work are summarized in Table 1 for thermoset angular composite parts. No matter whether the matrix is thermoset or thermoplastic, the spring-in angles are around 1° to 3° for a 90-degree V-shape or L-shape. However, spring-back behavior is also reported. As mentioned in the above sections, there are so many parameters, including intrinsic or extrinsic parameters, that could affect the angle deviation, and especially the interaction between these parameters may be not negligible. Furthermore, the warpage effect may always be present. Therefore, the effects obtained from different articles may not be so consistent, and controversial results may even be present.

## 3. Simulation Methods for Angle Deviations

The angle deviation of angular composite parts after manufacturing will cause fitting or other problems. To reduce this angle deviation in advance, one may modify the tool angle to compensate for this effect. Generally, the amount of modification is obtained by a trial-and-error approach or a “rule-of-thumb” approach. As indicated in the previous section, these approaches may be tedious, time-consuming, and expensive. Therefore, it is desired to predict the angle deviation of the designed angular composite parts via simulation methods, either analytic or numerical.

To simulate the process-induced deformation, there are not many differences between the warpage of flat laminates and the angle deviation of angular laminates, as the mechanisms to produce the residual stresses are similar. For a curing process, Wang et al. [2] summarized these mechanisms, including consolidation, inter-ply slip, tool interaction, relaxation, thermal expansion, chemical shrinkage, modulus development, thermal contraction, and free deformation. To model the general multiphysics phenomena, Baran et al. [3] pointed out thermokinetics, chemoreology for thermosets or thermoplastics, chemical shrinkage, and modeling of compaction. From the viewpoint of simulation, it may be better to separate these complicated mechanisms and modeling techniques into micro-scale and macro-scale effects. Micro-scale effects come from the constituent materials, the anisotropy of the fibers, chemical shrinkage of the resin, resin flow, and the degree of cure or crystallization. Macro-scale effects develop from layer anisotropy, including the mechanical and hygrothermal effects, lay-up sequence, tool materials, tool–part interaction, temperature gradient, and dimensional change of the part. Although Baran et al. [3] argued that the stresses at the micro-scale are self-equilibrating and do not create large deformations, the micro-scale effects are important in process-induced deformation. Most of the time, the micro-scale effects are just reflected in the properties of the composite layer by curve fitting or micromechanical calculation [45]. On the other hand, the macro-scale effects are mainly considered in the simulation.

As shown in Equation (2), three intrinsic parameters, including the thermal effect, hygroscopic effect, and shrinkage effect, could be considered as macro-scale effects to predict the angle deviation. Albert and Fernlund [7] used the $\Delta \theta_{Sk}$ or $\Delta \theta_{Sk}$ + $\Delta \theta_{CTE}$ in Equation (2) to predict the spring-in angles of C-shaped or L-shaped laminated parts made of carbon/epoxy prepregs. The lay-up may be unidirectional 0 degrees or quai-isotropic. To obtain the longitudinal and transverse CTEs for quai-isotropic laminates, methods such as classical lamination theory (CLT) combined with the micro-scale method [46] were used. Similar methods were also used to calculate the longitudinal shrinkage $\emptyset_{l}$ and the transverse shrinkage $\emptyset_{t}$ under the assumption that 2% of the resin volumetric shrinkage contributed to spring-in. However, their prediction, based on either the shrinkage effect or both the thermal and shrinkage effects, did not have good agreement with the experimental results for most cases considered. They claimed the difference mainly came from the flange/web warpage. Salomi et al. [39] argued that the shrinkage effect and the longitudinal CTE could be neglected for E-glass fabric/PP thermoplastic composite laminates. Only the thermal effect in Equation (2) was used with the assumption that the transverse CTE was linearly dependent on the temperature, in which two extra constants were needed and the glass transition temperature was considered. Even though there were two extra constants used to fit the experimental results, their original predictions were not good. By subtracting a corrective CTE from the transverse CTE in their equation, they could significantly improve the predictions, and they argued that this came from the CTE variations at the corners due to fiber wrinkling. By considering both the thermal effect and the shrinkage in Equation (2), Padovec et al. [19] claimed that good predictions were obtained for the spring-back behavior of L-shaped carbon/PPS thermoplastic parts by using CLT and 3D micromechanics equations [47], calculating those coefficients from the properties of fiber and matrix.

The above predictions were limited to a lot of simplifications and neglecting the interaction between the part and the tool. The widely used numerical method to estimate the angle deviation of angular composite parts is the finite element method. Generally, the finite element method can do both the macro-scale analysis and the micro-scale analysis in different simulation modules. In the macromechanical calculation, the elastic model [48,49], viscoelastic model [50,51,52,53,54], and path-dependent model [55,56] were three types of constitutive laws for flat composite laminate, with the corresponding model used to represent the curing process of the thermoset matrix. In the elastic model, a temperature-dependent cure-hardening instantaneous linear elastic (CHILL) model was commonly proposed to describe the resin modulus, $E_{m}$, as follows [57,58]:

(3) $$E_{m}=\left\{\begin{array}{l} E_{m}^{0}\\ E_{m}^{0}\\ E_{m}^{\infty } \end{array}\right.+\frac{T^{*}-T_{c1}}{T_{c2}-T_{c1}}\left(E_{m}^{\infty }-E_{m}^{0}\right)\;\;\;\begin{array}{l} T^{*}\leq T_{c1}\\ T_{c1}<T^{*}<T_{c2}\\ T_{c2}\leq T^{*} \end{array}$$

where $E_{m}^{0}$ and $E_{m}^{\infty }$ are the uncured and fully cured resin moduli, respectively. The critical temperatures at the onset and completion of the glass transition are denoted as $T_{C1}$ and $T_{C2}$, respectively. The glass transition temperature $T_{g}$ and the temperature *T* of the resin are used to define $T^{*}=T_{g}-T$. The viscoelastic constitutive models accounted for the stress relaxations during the polymerization process. The relaxed modulus of the thermoset matrix [51] can be approximated using the Prony series expressed as follows [59,60]:

(4) $$E_{m}\left(\varphi ,\;T,\;t\right)=E_{m}^{rel}+\left(E_{m}^{rel}-E_{m}^{unrel}\right)\overset{n}{\underset{i}{\sum }}w_{i}\exp\left[\frac{-\xi \left(\varphi ,T\right)}{\tau \left(\varphi \right)}\right]$$

where $E_{m}^{rel}$ and $E_{m}^{unrel}$ are the fully relaxed and unrelaxed resin moduli, respectively. The current time is denoted as *t*, and the weight fitting factor is $w_{i}.$ The discrete relaxation time $\tau \left(\varphi \right)$ is a function of the degree of cure φ, and ξ is the current reduced time that is a function of the degree of cure and the temperature. The path-dependent model [55,56] was considered a simplified version of the viscoelastic model. In this model, the moduli of the resin are constants during the rubbery state and the glassy state, and the former values may be two orders of magnitude smaller than the latter. The modulus could be written as follows [55,56]:

(5) $$E_{m}=\left\{\begin{array}{l} E_{m}^{r}\\ E_{m}^{g} \end{array}\right.\;\;\;\begin{array}{l} T^{*}\leq 0\\ T^{*}>0 \end{array}$$

The above three types of constitutive laws were commonly used to simulate the process-induced deformation of flat composite laminates. However, the following summary focuses on the numerical methods to predict the angle deviation of angular composite parts. Johnston et al. [57] presented a plane strain chemical-thermomechanical (CTM) finite element model to simulate the process-induced deformation of L-shaped laminates. A temperature-dependent CHILE constitutive model [58], Equation (3), was used to describe the behavior of the composite matrix resin, and self-consistent micromechanics models (SC) [48] were applied to obtain the mechanical properties of the composite layers, including the thermal expansion and cure shrinkage. The tool–part interaction was modeled by a cure-hardening elastic shear layer remaining intact until the tool was removed [31,58]. They predicted a 1.79° spring-in angle for [0]_24_ lay-ups with good agreement with the experimental angle of 1.77°. However, a large error occurred between the predicted and measured spring-in values for [90]_24_ lay-ups, and they thought that the reason was the overly simplified model of the tool/part interaction. A simple two-step finite element model for the manufacturing process was developed by Ersoy et al. [27] to predict the spring-in angle of C-section parts made of AS4/8552 thermoset composite. The mechanical properties of the unidirectional composite needed in ABAQUS were obtained via finite element-based micromechanics (FEM) [61] according to the rubbery state for the first step before vitrification and the glassy state for the second state after vitrification. The tool was not modeled by just assigning sliding boundary conditions. Their correlation with the experimental values was good for both the unidirectional and cross-ply parts. The experimental trend of decreasing spring-in angles with increasing thickness was predicted. In addition to the thermal expansion along and across the fibers, Wiersma et al. [62] used the finite element package DIEKA [63], including an inhomogeneous fiber/matrix distribution through the thickness, an inhomogeneous heat distribution during the cure cycle, and the difference in thermal expansion between the tool and the part. However, the spring-in angle of L-shaped parts could not be predicted completely by their method. They suggested that the complete cure cycle and a viscoelastic model [50,64] for composites may be necessary. Clifford et al. [65] considered both the anisotropy and viscoelasticity in their macro-scale finite element model to simulate the cooling process of V-shaped parts made of non-crimp carbon fiber fabric reinforced epoxy. Since the micro-scale model was not included, they over-predicted the part deformation.

Bapanapalli and Smith [15] used a simplified four-layer finite element model, in which the first layer of the part, as shown in Figure 3, was affected by the tool stretching by increasing its longitudinal CTE and thickness. These two values were obtained empirically. In addition, the cure shrinkage was considered in the model by increasing the through-thickness CTE until an agreement with the unidirectional L-shaped parts was achieved. Their prediction had 2–11% errors in the spring-in angles for L-shaped cross-ply laminates and 6–17% errors for U-shaped cross-ply laminates. A semi-empirical approach with three steps was proposed by Fiorina et al. [66] to predict the spring-in value of aircraft wing box ribs made of carbon/epoxy laminates. In the first step, a specific CTE of the first layer of the laminate was identified numerically from the experimental results [67,68] and combined with an interface to give the in-plane stress gradient through the laminate thickness, as shown in Figure 3. In the second step, the spring-in behavior of the full structure was modeled using shell elements and a linear elastic model. In the third step, local 3D models of the important sections for spring-in were made of the laminate only with the first ply and without the tool or interface. By means of this method, their predictions had a reasonable agreement with the normalized spring-in angles of L-shaped parts. Nasir et al. [14] included an interface layer between the laminate and the part and split the first layer of the laminate into the corner section and the flange section, in which they possessed different longitudinal CTEs. By considering only the cool-down phase of the entire curing cycle and thermomechanical (TM) behavior, they could have 1.02–1.27% errors in the spring-in warpage of L-shaped parts with 30° to 90° tool angles, as the CTE of the corner section was adjusted. Two modules of the software PAM were used by Bellini et al. [30] to estimate the spring-in value of L-shaped parts. The first module, PAM-RTM, implemented a thermochemical (TC) model to determine the temperature field and the cure degree trend in the laminate, and these results were used in the next step. The second module, PAM-Distortion, was a thermomechanical model calculating the residual stresses and distortions of laminates due to the manufacturing process. The part was assumed to be a frictionless contact with the tool. Their simulation results had 3–17% errors with the experimental spring-in values for L-shaped parts with different lay-ups and thicknesses. They also predicted that only the lay-up influenced the spring-in angles, while the part thickness and the corner radius did not affect those values. 

Fernlund et al. [31] used the software COMPRO to integrate a deformation model, a heat transfer model, and a resin flow model, and they applied empirical functions for the property development of the composites. They focused on using different types of shear layer materials, either soft or hardening, between the part and the tool to represent the tool surface conditions. In most cases, either L-shaped or C-shaped parts had accurate results with the experimental values. However, there were larger errors in some cases, of which either thermal expansion or resin cure shrinkage may be the source. Patil et al. [69] considered the resin shrinkage, degree of cure, and the difference between the CTEs of tools and parts by using ANSYS Composite Cure Simulation with a structure cure module input from a semi-empirical thermal cure module, which considered a cure kinetic model [70]. A frictionless support was applied at the outer surface of the part to represent the tool. Even though they had underestimated the spring-in angles, their results were reasonable with the experimental values for L-shaped laminate parts. Liu et al. [71] used a micro-scale finite element model [72] to characterize the equivalent mechanical properties of unidirectional composites based on a simplified constitutive model of the resin matrix considering the stress relaxation, which was based on the CHILE model and the path-dependent model [55,56]. Then, a macro-scale finite element model including the part, the tool, and the interface friction between them was used to simulate the curing process, including the glassy and rubbery states. Their predictions for L-shaped parts made of carbon fiber-reinforced bismaleimide composites had 6–13% errors in the spring-in angles for most cases. Qiao et al. [73] considered a 3D numerical model based on a modified path-dependent model to predict the spring-in angles of L- and U-shaped composite parts. A simplified self-consistent micromechanics model was utilized to predict the properties of the composites. When the shear layer was used between the tool and the part, the prediction errors were under 9.64% for both the L-shaped and U-shaped parts. Kim et al. [74] evaluated the spring-in angles of L-shaped plain woven composite structures without comparison with the experimental results. The viscoelastic constitutive model with the relaxed modulus of the thermoset matrix, Equation (4), was used, and the effective material properties of the plain woven composites were predicted using a self-consistent micromechanics model for a unit cell. The tool–part interaction was represented as a fixed boundary condition at the corner. The above numerical methods used to predict the angle deviation of angular composite parts are compared and summarized in Table 2. 

For thermoplastic composite materials, a spring-in model [75,76] using CLT with an extra thickening term that may be contributed from thermal expansion, in-plane residual stress, etc., was applied by Jain et al. [40] to iteratively solve the mid-plane strains and curvatures. The typical properties of a lamina were determined from the micromechanics relations. Then, the spring-in angle could be predicted from the enclosed angle, mid-plane radius, and mid-plane curvature. A good agreement was found for the aileron rib manufactured from carbon fiber fabric/PEI thermoplastic composites. Han et al. [42] applied ABAQUS to simulate the thermoforming and demolding processes by using the temperature-dependent orthotropic elastic and both the temperature and strain rate-dependent plastic behavior of the composites obtained via micromechanical theory. Their predictions agreed with the trend from the tool temperatures, in which higher spring-in angles were derived from higher tool temperatures. However, the predicted spring-in angles were much lower than the experimental results. Padovec et al. [19] used ABAQUS to analyze the thermo-elastic strains for L-shaped carbon/PPS thermoplastic parts by using CLT and 3D micromechanics equations, and reasonable agreement was obtained between the simulated spring-back values and the experimental results.

When Equation (2) is used to predict the angle deviation of angular composite parts, the important parameters are the coefficients and the change in temperature and moisture. These are considered intrinsic parameters. In most cases, the coefficients can be calculated in certain ways, such as micromechanical theory and/or classical lamination theory, to account for the lay-up, thickness, and tool angle. However, the extrinsic parameters and the interaction between the tool and the part are not considered. Hence, predictions by Equation (2) are generally not good. For the finite element methods, they can include the lay-ups, the thermo-mechanical behavior, and the interaction between the tool and the part. However, their accuracy still depends on the proper choice of the properties of the composite, especially the effect of the cure shrinkage of the resin. Even though some researchers seemed to consider both the micromechanical situation and the marcomechanical behavior in their methods, the transition between these two stages is still a challenge. In addition, thermoset and thermoplastic resins may have different behaviors in the manufacturing process, which should be considered in the micromechanical stage.

## 4. Conclusions

Fiber-reinforced polymer matrix composites are popular candidates for weight-critical structural components applied in various industries because of their specific properties. For thermoset matrix composites, autoclave curing is the main process used to solidify the prepregs into the desired shape. For thermoplastic matrix composites, thermoforming may be a popular process with which to make semi-finished products into final products. During these processes, residual stresses may be created, resulting in warpage in flat structures and angle changes in angular sections. These shape distortions may create fitting mismatch problems. The present paper focuses on the angle deviation of angular composite laminates made of either thermoset or thermoplastic matrix. Depending on the positive or negative angle deviation, spring-back or spring-in behavior is observed. There are many parameters, including intrinsic or extrinsic parameters, that can affect the angle deviation. In the first part of the present paper, the experimental results are summarized, and the spring-in effect is presented in most cases. Generally, the spring-in angle is linear and inversely proportional to the part angle, and the angle deviations are reduced with the increase in the part thickness. The lay-up sequence is indicated to have a significant influence. The corner radius, flange size, and part shape may have minor or no obvious effect. As for the extrinsic parameters, the tool materials have no consistent trend, and smoother surfaces may have larger spring-in angles. A two-stage cure cycle may result in larger spring-in angles than a one-stage cycle. In the second part, prediction methods such as a simple equation and finite element methods are compared to indicate the considered parameters. When using a simple equation, most researchers did not achieve good predictions of the angle deviations. When using the finite element methods, even though different commercial software packages were used, good agreement could be obtained. The crucial differences may be dependent on the input properties of the composite and the constituents. In addition, micromechanical theories are important to estimate the input properties before the application of marcomechanical analysis.

## Figures and Tables

**Table 1:** Parameter effects from experiments for thermoset angular composites.

	Spring-in			Spring-back	
	Increase	Little	Decrease	Increase	Decrease
Part angle			[14,23]		[24]
Part thickness	[28]	[29]	[7,20,25,26,27,44]		[24,33]
Flange size	[7]			[24]	
Corner radius		[29,30]			
Lay-up sequence (cross-ply, quasi-isotropic)	[7,27,30,31]	[29]			[33]
Tool material	[7] (Al)				[33] (Invar)
Tool surface (release agent + FEP)	[31]		[7,31]		
Cure cycle (two-stage)	[7,31]			[33]	

**Table 2:** Comparison of numerical methods applied for thermoset angular composites.

Ref.	Resin	Micro.	Macro.	Interface	Shape	Error
[40]	Equation (3)	SC	CTM	Shear layer (tool)	L, U	Good, Bad
[13]	Equation (5)	FEM	CTM	Sliding (no tool)	C	Good
[46]	Equation (4)		CTM	No slip (tool)	L	Bad
[51]	Equation (4)		CTM	Contact (tool)	V	Bad
[52]			CTM	Shear layer (no tool)	L, U	Reasonable
[53]			Global + Local	Interface (tool)	Rib	Reasonable
[8]			TM	Interface (tool)	L	Good
[16]	Equation (5)		TC + TM	Contact (tool)	L, U	Reasonable
[17]			CTM	Shear layer (tool)	L, C	Good, Bad
[56]			TC + TM	Frictionless support	L	Reasonable
[71]	Equations (3) and (5)	FEM	CTM	Friction (tool)	L	Reasonable
[73]	Equation (5)	SC	CTM	Shear layer (tool)	L, U	Good
[74]	Equation (4)	SC	CTM	Fixed corner (no tool)	L	

## Data Availability

Not applicable.
